# Editorial: Bacteria-host interactions: from infection to carcinogenesis

**DOI:** 10.3389/fcimb.2025.1634299

**Published:** 2025-06-11

**Authors:** Marco A. Hernández-Luna

**Affiliations:** Department of Medicine and Nutrition, Division of Health Science, University of Guanajuato, León, Mexico

**Keywords:** bacteria-host interaction, carcinogenesis, tumoral microbiome, bacterial infection, viral infection

An increasing number of microorganisms are being associated with the development and progression of cancer. The advent of techniques such as Next Generation Sequencing (NGS) has enabled the study of the tumor microbiome, opening a new field of research aimed at understanding the role of microorganisms—such as viruses and bacteria—in carcinogenesis.

This Research Topic, titled “*Bacteria–Host Interactions: From Infection to Carcinogenesis*,” includes twelve publications: eight original research articles and four review articles, all of which describe the mechanisms through which microorganisms contribute to carcinogenesis.

Human papillomavirus (HPV) is a well-known infectious agent closely associated with various types of cancer. Persistent infection with HPV types 16 and 18 leads to the production of E6 and E7 viral proteins, which inactivate tumor suppressor genes such as p53 and pRb. This alters the cell cycle and promotes uncontrolled cell proliferation. While the mechanisms by which HPV induces carcinogenesis have been extensively studied, its impact on the vaginal microbiome remains less understood. In this regard, Li and Wu explored the association between HPV infection, cervical lesion severity, and the composition of the vaginal microbiome, proposing that microbial changes may influence cervical cancer progression.

*Helicobacter pylori* (*H. pylori*) has been identified as a major risk factor for gastric cancer (GC). Chronic infection results in persistent gastric mucosal inflammation, which can evolve into gastric atrophy, intestinal metaplasia, dysplasia, and, eventually, cancer. However, the mechanisms enabling this bacterium to colonize tumor tissue are not yet fully understood. Liu et al. investigated the relationship between progranulin (PGRN), a multifunctional protein dysregulated in various cancers, and *H. pylori* colonization. Their study describes how increased PGRN expression—induced by *H. pylori*—inhibits autophagy via the mTOR pathway, thereby facilitating bacterial internalization. These findings may contribute to the development of new therapeutic targets for reducing the risk of *H. pylori*–associated gastric cancer.

Moreover, *H. pylori* has been shown to promote carcinogenesis through other mechanisms, including mitochondrial DNA (mtDNA) damage. Shahi et al. published a review examining how *H. pylori* compromises mitochondrial genomic integrity by affecting the base excision repair mechanisms involving proteins such as POLG, TFAM, and MGME1. These alterations may lead to mtDNA deletions, increasing susceptibility to chronic inflammatory diseases linked to bacterial infections. The review also discusses how mtDNA damage and repair influence immune responses during *H. pylori* infection.

*H. pylori* has likewise been associated with programmed cell death (PCD) mechanisms, including apoptosis, autophagy, and necroptosis. Lin et al. reviewed how *H. pylori* activates PCD pathways. The bacterium interferes with apoptosis, leading to gastric tissue damage, and can also promote pyroptosis via the NLRP3 inflammasome pathway, which contributes to gastric tumor growth. The review further addresses how *H. pylori*–induced inhibition of autophagy could support carcinogenesis. Additional forms of cell death, such as necroptosis and ferroptosis, are also discussed in the context of bacterial involvement.

The available experimental models present significant challenges for understanding bacterial infections like that caused by *H. pylori*. Both *in vitro* and *in vivo* models are essential tools in this field. *In vitro* models provide a controlled environment to study bacteria–host interactions, while *in vivo* models offer insights into the progression of infection in a whole organism. However, the degree to which these models accurately replicate human infection remains a topic of debate. Patil et al. reviewed various infection models for *H. pylori* and discussed their applications in vaccine and drug development.

NGS technologies have allowed researchers to analyze changes in the tumor microbiome, revealing potential links between microbiota composition and cancer progression. Liu et al. investigated correlations between body mass index in GC patients, tumor-associated microbiota, and prognosis. Their findings suggest that the presence of bacteria from the genus *Abiotrophia*, which are associated with immune evasion, may promote tumor progression. This opens the possibility of antibiotic-based cancer therapies that target specific bacteria within the tumor microenvironment.

Oral cancer is a malignancy affecting tissues in the oral cavity, including the lips, tongue, gums, palate, and buccal mucosa. It has been strongly linked to tobacco and alcohol use, as well as infections such as HPV. In recent years, bacteria like *Fusobacterium nucleatum* (*F. nucleatum*) have also been associated with various gastrointestinal cancers, including those of the oral cavity. Although the precise mechanisms remain unclear, Selvaraj et al. reported that *F. nucleatum*’s adhesion to and invasion of oral epithelial cells enhances cell migration and invasion, potentially favoring metastasis in gastrointestinal tumors.

The oral microbiota comprises over 700 species of commensal bacteria, with the genus Streptococcus being among the most abundant. Its role in carcinogenesis is not fully understood. Inui et al. used an *in vitro* model found that *Streptococcus mitis* have a protective effect. The bacterium inhibited cell proliferation in oral squamous cell carcinoma (OSCC) by regulating the cell cycle, suggesting that certain commensal bacteria may play a role in controlling carcinogenesis and could be explored as therapeutic agents for oral cancers.

Global Transcriptomic Network Analysis allows large-scale exploration of gene interactions by integrating gene expression profiles. This approach enables the identification of patterns and contributes to the discovery of novel therapeutic targets. Using this method, Otálora-Otálora et al. found that neoplasms such as stomach and lung cancers share similarities in the expression of transcription factors regulating genes for receptors that interact with viruses such as HTLV-1, HPV, EBV, and SARS-CoV-2. Their research furthers our understanding of how microorganisms may influence cancer development and provides foundational data for improving diagnosis and treatment strategies for gastrointestinal and lung cancers.

Vimentin plays a pivotal role in cancer progression, being associated with epithelial–mesenchymal transition (EMT) and metastasis. Overexpression of vimentin in tumor cells enhances cell motility, invasiveness, and resistance to apoptosis. The factors driving vimentin upregulation remain unclear. In this context, Jang et al. reported that *Staphylococcus aureus* (*S. aureus*) induces vimentin expression via the TLR2 signaling pathway, facilitating its internalization into keratinocytes. Their study not only highlights vimentin as a potential therapeutic target for managing *S. aureus* infections, but also suggests that pathogenic microorganisms may promote tumor progression by enhancing vimentin expression.

Finally, an increasing number of bacterial species are being implicated in the carcinogenic process. Solomon et al. reviewed the role of *Ehrlichia chaffeensis* (*E. chaffeensis*), an intracellular bacterium that evades the host immune system and activates carcinogenic signaling pathways such as Wnt, Notch, and Hedgehog via Short Linear Motifs (SLiMs). Activation of these pathways by *E. chaffeensis* results in apoptosis inhibition, enhanced cell survival, and increased proliferation. Understanding the mechanisms by which *E. chaffeensis* modulates these cellular pathways could facilitate the development of new anticancer therapies and the identification of novel therapeutic targets.

